# Gross total resection and survival outcomes in elderly patients with spinal chordoma: a SEER-based analysis

**DOI:** 10.3389/fonc.2023.1327330

**Published:** 2024-01-30

**Authors:** John Pham, Elias Shaaya, Ben Rhee, Anna Kimata, Evrim E. Ozcan, Katie M. Pham, Tianyi Niu, Patricia Sullivan, Ziya L. Gokaslan

**Affiliations:** Department of Neurosurgery, Rhode Island Hospital, The Warren Alpert Medical School of Brown University, Providence, RI, United States

**Keywords:** gross total resection, spinal chordoma, elderly population, survival outcomes, SEER

## Abstract

**Objective:**

The association between aggressive resection and improved survival for adult spinal chordoma patients has not been well characterized in the geriatric population. Thus, the present study aimed to elucidate the relationship between gross total resection (GTR) and survival outcomes for patients across different age groups.

**Methods:**

The authors isolated all adult patients diagnosed with spinal chordoma from the 2000-2019 Surveillance, Epidemiology, and End Results database and divided patients into three surgical subgroups: no surgery, subtotal resection (STR), and GTR. Kaplan-Meier curves with a log-rank test were used to discern differences in overall survival (OS) between surgical subgroups. Univariate and multivariate analyses were used to identify prognostic factors of mortality.

**Results:**

There were 771 eligible patients: 227 (29.4%) received no surgery, 267 (34.6%) received STR, and 277 (35.9%) received GTR. Patients receiving no surgery had the lowest 5-year OS (45.2%), 10-year OS (17.6%), and mean OS (72.1 months). After stratifying patients by age, our multivariate analysis demonstrated that patients receiving GTR aged 40-59 (HR=0.26, CI=0.12-0.55, p<0.001), 60-79 (HR=0.51, CI=0.32-0.82, p=0.005), and 80-99 (HR=0.14, CI=0.05-0.37, p<0.001) had a lower risk of mortality compared to patients undergoing no surgery. The frequency of receiving GTR also decreased as a function of age (16.4% [80-99 years] vs. 43.2% [20-39 years]; p<0.001), but the frequency of receiving radiotherapy was comparable across all age groups (48.3% [80-99 years] vs. 45.5% [20-39 years]; p=0.762).

**Conclusion:**

GTR is associated with improved survival for middle-aged and elderly patients with spinal chordoma. Therefore, patients should not be excluded from aggressive resection on the basis of age alone. Rather, the decision to pursue surgery should be decided on an individual basis.

## 1 Introduction

Chordomas are rare, primary malignant osseous tumors that arise from primitive notochordal crest cells. While these lesions are located throughout the neuraxis, over 60% of chordomas arise from the sacrococcygeal region and the mobile spine (1). Due to their locally aggressive and invasive nature as well as their proximity to the spinal cord and functional nerve roots, spinal chordomas have a poor natural history associated with significant morbidity and mortality (2, 3). Since chordomas have high rates of local recurrence and are classically resistant to radiotherapy and chemotherapy, en bloc resection (with marginal or wide margins) or gross total resection (GTR) with adjuvant proton or photon radiotherapy remain the preferred treatment options to optimize patient outcomes (4–6).

From a technical perspective, en bloc surgical resection generally requires larger surgeries, with greater morbidity for patients. Mobile spine chordomas may require anterior and posterior combination approaches in order to achieve en bloc resection. These staged surgical procedures can result in higher blood loss, longer anesthesia times and greater bodily stress. For this reason, a patient’s preoperative health must be considered in the preoperative planning phase.

Recent advances in surgical techniques and general anesthesia, better management of medical comorbidities, and the general aging of high-income countries have increased the demand for spinal surgery in elderly patients (7, 8). However, there is reluctance amongst spine surgeons to operate on older patients due to a higher expected rate of postoperative complications as well as reduced functional reserves among the elderly (9). Consequently, older patients with chordoma are much less likely to receive surgery than younger patients (10). However, recent studies have shown improvements in neurologic function and quality of life (QoL) for geriatric patients undergoing resection of spinal neoplasms, including meningiomas and metastatic tumors (11–13).

For primary malignant tumors of the spine like chordoma, the goal of resection is not only to prevent neurologic decline from tumor progression, but also to extend survival. Notably, there is a dearth of knowledge on the association between extent of resection and survival outcomes in geriatric patients with spinal chordoma. Previous work has established that the incidence of spinal chordoma increases with age, with the highest incidence in the eighth decade of life, and the median age of diagnosis is approximately 60 years (10, 14, 15). Despite this, the available literature provides few descriptions of geriatric patients surgically treated for spinal chordoma, consisting mostly of case reports or underpowered single and multi-institutional studies dominated by younger subjects (4–6, 16).

As geriatric patients are frequently omitted from spinal chordoma clinical studies, registry data can provide key insights into treatment patterns and survival outcomes for this age group. The Surveillance, Epidemiology, and End Results (SEER) database of the National Cancer Institute provides population-based data related to cancer diagnoses, treatment, and survival information for approximately 30% of the United States (U.S.) population (17). Importantly, it allows for a nationally representative cohort of the geriatric U.S. population. In this study, we aim to elucidate the relationship between GTR and survival outcomes for adult patients with spinal chordoma across different age groups. Additionally, we seek to determine whether patient age influences the frequency that GTR and radiotherapy is performed.

## 2 Materials and methods

### 2.1 Study population

We queried all patients diagnosed with spinal chordoma from the SEER Research Plus Dataset, 17 Registries (2000–2019), using SEER*Stat software (version 8.4.0.1). Due to the anonymized nature of the database, this study was exempt from Brown University Institutional Review Board approval and was performed in accordance with the Declaration of Helsinki of 1975 (as revised in 2000). The International Classification of Diseases for Oncology Third Edition was used to identify histologically confirmed cases of chordoma (9370/3: Chordoma, NOS [not otherwise specified]; 9371/3: Chondroid Chordoma; 9372/3: Dedifferentiated Chordoma). Patients were excluded if: a) their primary site code was not C41.2 (vertebral column) or C41.4 (pelvic bones, sacrum, coccyx, and associated joints) (n=921); b) it was unknown if surgery was performed or they had an unknown extent of resection (n=35); c) they had an unknown survival time (n=2); or d) they were under 20 years old or had an unknown age (n=18). Finally, a total of 771 patients with spinal chordoma were included in this study.

### 2.2 Study variables

Independent covariates included age at diagnosis, sex, marital status, race, primary site, disease stage, histological type, tumor size, and year of diagnosis, as well as treatment information such as surgery, radiotherapy, and chemotherapy. Patients were divided into four groups according to age: 20-39 years, 40-59 years, 60-79 years, and 80-99 years.

We trichotomized patients into 3 surgical groups based on their extent of resection: no surgery, subtotal resection (STR), and GTR. In this study, STR refers to SEER surgery codes [RX Summ—Surg Prim Site (1998+)] 15, 19, 25, and 26. GTR encompasses surgery codes 30, 40, 41, 42, 50, 52, 53, and 54. These classifications are consistent with previous SEER-based studies on spinal chordoma (18, 19). Detailed information on SEER surgery code definitions can be found in **Supplementary Table 1**. To account for advancements in the diagnosis and treatment of spinal chordoma over time, the year of diagnosis was included as a covariate stratified into 4 groups (2000-2004, 2005-2009, 2010-2014, and 2015-2019).

The primary outcome was overall survival (OS), which considers death from any cause an event, starting from the date of diagnosis. Patients still alive at the end of follow-up were censored.

### 2.3 Statistical analysis

Descriptive statistics were calculated for all variables. Chi-square tests were performed to detect significant associations between categorical variables. The likelihood of receiving GTR or radiotherapy after adjusting for demographic and clinical covariates was evaluated using a multivariable logistic regression model. The results were presented as an odds ratio (OR) with 95% confidence intervals (CI). Kaplan-Meier curves were generated to estimate the impact of surgical status on mean OS, 5-year OS, and 10-year OS, and differences between the curves were inferentially tested using the log-rank test. Univariate and multivariate Cox proportional hazard regression models were utilized to assess the predictive performance of extent of resection on all-cause mortality after adjusting for demographic and clinical covariates. Their effects were presented as a hazard ratio (HR) with 95% CI. All statistical analyses were performed using SPSS 28 software (IBM Corp., Armonk, NY). A two-tailed p value <0.05 was considered statistically significant.

## 3 Results

### 3.1 Patient characteristics and patterns of care

**Figure 1:**
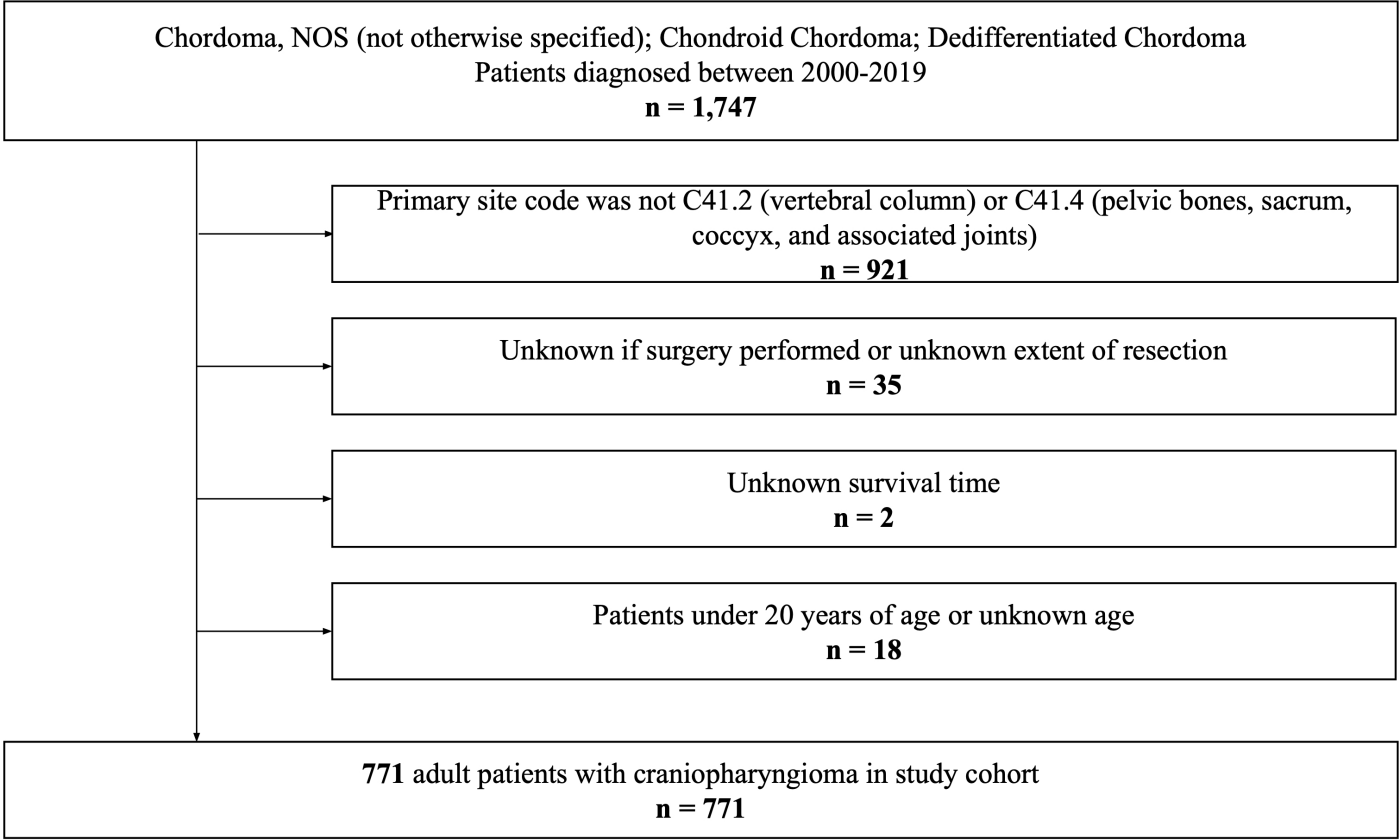
Inclusion and exclusion criteria for study enrollment.

After excluding 976 ineligible patients, a total of 771 adult patients diagnosed with spinal chordoma between 2000 and 2019 from the SEER database were included (**Figure 1**). Of this cohort of eligible patients, approximately 60% were elderly: 335 were in the 60-79 age cohort (43.5%) and 116 were in the 80-99 age cohort (15.0%). Summaries of baseline patient characteristics grouped by extent of resection and by age at diagnosis are shown in **Tables 1**, **2**, respectively.

**Table 1:** Clinical and Demographic Characteristics of Spinal Chordoma Patients Undergoing No Surgery, STR, or GTR.

Characteristic	Total No. (%)	No Surgery (%)	STR (%)	GTR (%)	P value
Total	771 (100%)	227 (29.4%)	267 (34.6%)	277 (35.9%)	
Age at Diagnosis					**P<0.001**
20-39	88 (11.4%)	11 (4.8%)	39 (14.6%)	38 (13.7%)	
40-59	232 (30.1%)	47 (20.7%)	85 (31.8%)	100 (36.1%)	
60-79	335 (43.5%)	96 (42.3%)	119 (44.6%)	120 (43.3%)	
80-99	116 (15.0%)	73 (32.2%)	24 (9.0%)	19 (6.9%)	
Sex					P=0.452
Male	465 (60.3%)	135 (59.5%)	155 (58.1%)	175 (63.2%)	
Female	306 (39.7%)	92 (40.5%)	112 (41.9%)	102 (36.8%)	
Marital Status					**P<0.001**
Married	448 (58.1%)	108 (47.6%)	165 (61.8%)	175 (63.2%)	
Single	134 (17.4%)	35 (15.4%)	46 (17.2%)	53 (19.1%)	
Divorced/Separated	68 (8.8%)	23 (10.1%)	22 (8.2%)	23 (8.3%)	
Widowed	79 (10.2%)	45 (19.8%)	20 (7.5%)	14 (5.1%)	
Unknown	42 (5.4%)	16 (7.0%)	14 (5.2%)	12 (4.3%)	
Race					**P=0.045**
White	663 (86.0%)	186 (81.9%)	238 (89.1%)	239 (86.3%)	
Black	27 (3.5%)	6 (2.6%)	10 (3.7%)	11 (4.0%)	
Other	81 (10.5%)	35 (15.4%)	19 (7.1%)	27 (9.7%)	
Primary Site					**P<0.001**
Vertebral Column	319 (41.4%)	71 (31.3%)	163 (61.0%)	85 (30.7%)	
Pelvic bones, sacrum, coccyx, and associated joints	452 (58.6%)	156 (68.7%)	104 (39.0%)	192 (69.3%)	
Disease Stage					**P<0.001**
Localized	335 (43.5%)	85 (37.4%)	143 (53.6%)	107 (38.6%)	
Regional	309 (40.1%)	70 (30.8%)	95 (35.6%)	144 (52.0%)	
Distant	79 (10.2%)	38 (16.7%)	19 (7.1%)	22 (7.9%)	
Unstaged	48 (6.2%)	34 (15.0%)	10 (3.7%)	4 (1.4%)	
Histological Type					P=0.221
Chordoma, NOS	745 (96.6%)	220 (96.9%)	259 (97.0%)	266 (96.0%)	
Conventional chordoma	16 (2.1%)	2 (0.9%)	5 (1.9%)	9 (3.2%)	
Dedifferentiated chordoma	10 (1.3%)	5 (2.2%)	3 (1.1%)	2 (0.7%)	
Tumor Size					**P<0.001**
<5 cm	180 (23.3%)	35 (15.4%)	79 (29.6%)	66 (23.8%)	
5-10 cm	262 (34.0%)	75 (33.0%)	80 (30.0%)	107 (38.6%)	
>10 cm	129 (16.7%)	46 (20.3%)	28 (10.5%)	55 (19.9%)	
Unknown	200 (25.9%)	71 (31.3%)	80 (30.0%)	49 (17.7%)	
Radiotherapy					P=0.183
Yes	368 (47.7%)	113 (49.8%)	135 (50.6%)	120 (43.3%)	
No	403 (52.3%)	114 (50.2%)	132 (49.4%)	157 (56.7%)	
Chemotherapy					**P<0.001**
Yes	39 (5.1%)	25 (11.0%)	4 (1.5%)	10 (3.6%)	
No	732 (94.9%)	202 (89.0%)	263 (98.5%)	267 (96.4%)	
Year of Diagnosis					P=0.497
2000-2004	137 (17.8%)	38 (16.7%)	57 (21.3%)	42 (15.2%)	
2005-2009	168 (21.8%)	46 (20.3%)	61 (22.8%)	61 (22.0%)	
2010-2014	215 (27.9%)	64 (28.2%)	72 (27.0%)	79 (28.5%)	
2015-2019	251 (32.6%)	79 (34.8%)	77 (28.8%)	95 (34.3%)	

P value <0.05 are shown in bold.

STR, subtotal resection; GTR, gross total resection; NOS, not otherwise specified.

Percentages may not add up to 100 because of rounding.

**Table 2:** Clinical Characteristics of Spinal Chordoma Patients According to Age at Diagnosis.

Age at Diagnosis	20-39 (%)	40-59 (%)	60-79 (%)	80-99 (%)	P value
Total	88 (100%)	232 (100%)	335 (100%)	116 (100%)	
Primary Site					P=0.426
Vertebral Column	43 (48.9%)	98 (42.2%)	132 (39.4%)	46 (39.7%)	
Pelvic bones, sacrum, coccyx, and associated joints	45 (51.1%)	134 (57.8%)	203 (60.6%)	70 (60.3%)	
Disease Stage					P=0.670
Localized	40 (43.5%)	99 (42.7%)	153 (45.7%)	43 (37.1%)	
Regional	32 (36.4%)	91 (39.2%)	134 (40.0%)	52 (44.8%)	
Distant	9 (10.2%)	26 (11.2%)	28 (8.4%)	16 (13.8%)	
Unstaged	7 (8.0%)	16 (6.9%)	20 (6.0%)	5 (4.3%)	
Histological Type					P=0.303
Chordoma, NOS	83 (94.3%)	221 (95.3%)	327 (97.6%)	114 (98.3%)	
Chondroid chordoma	3 (3.4%)	8 (3.4%)	5 (1.5%)	0 (0.0%)	
Dedifferentiated chordoma	2 (2.3%)	3 (1.3%)	3 (0.9%)	2 (1.7%)	
Tumor Size					P=0.073
<5 cm	31 (35.2%)	61 (26.3%)	69 (20.6%)	19 (16.4%)	
5-10 cm	29 (33.0%)	73 (31.5%)	120 (35.8%)	40 (34.5%)	
>10 cm	9 (10.2%)	35 (15.1%)	61 (18.2%)	24 (20.7%)	
Unknown	19 (21.6%)	63 (27.2%)	85 (25.4%)	33 (28.4%)	
Radiotherapy					P=0.762
Yes	40 (45.5%)	117 (50.4%)	155 (46.3%)	56 (48.3%)	
No	48 (54.5%)	115 (49.6%)	180 (53.7%)	60 (51.7%)	
Chemotherapy					P=0.182
Yes	7 (8.0%)	11 (4.7%)	12 (3.6%)	9 (7.8%)	
No	81 (92.0%)	221 (95.3%)	323 (96.4%)	107 (92.2%)	

P value <0.05 are shown in bold.

STR, subtotal resection; GTR, gross total resection; NOS, not otherwise specified.

Percentages may not add up to 100 because of rounding.

**Figure 2:**
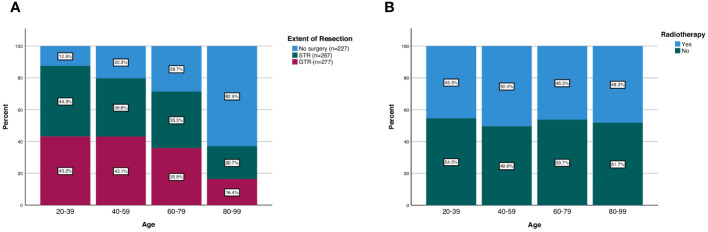
Patterns of **(A)** extent of resection and **(B)** radiotherapy treatment in spinal chordoma patients stratified by age group.

Among all patients, 277 received no surgery (29.4%), 267 received STR (34.6%), and 277 received GTR (35.9%). The frequency of receiving GTR decreased as a function of age; 43.2% of patients aged 20-39 received GTR compared to 16.4% of patients aged 80-99 (p<0.001; **Figure 2**). Similarly, the frequency of receiving any surgery (STR or GTR) decreased as a function of age; 87.5% of patients aged 20-39 received surgery compared to 37.1% of patients aged 80-99 (p<0.001; **Figure 2**). In our multivariable logistic regression analysis with GTR as the dependent variable, patients aged 80-99 were 0.23 times as likely (95% CI 0.11-0.47, p<0.001) to receive GTR compared to patients aged 20-39 (**Table 3**). However, there was no statistical difference in the likelihood of receiving GTR between patients aged 20-39 (reference) and patients aged 40-59 (OR 0.88; 95% CI 0.51-1.51, p=0.643) or patients aged 60-79 (OR 0.59; 95% CI 0.35-1.02, p=0.057) (**Table 3**). Other variables significantly predictive of receiving GTR included primary site (p<0.001) and disease stage (p<0.001) (**Table 3**).

**Table 3:** Logistic Regression of Spinal Chordoma Patients (n=771), Demonstrating Clinical and Demographic Factors that were Predictive of Undergoing GTR.

Characteristic	OR (95% CI)		P value	
Age at Diagnosis			**P<0.001**	
20-39	Reference			
40-59	0.88 (0.51-1.51)		P=0.643	
60-79	0.59 (0.35-1.02)		P=0.057	
80-99	0.23 (0.11-0.47)		P<0.001	
Sex				
Male	Reference			
Female	0.88 (0.63-1.23)		P=0.459	
Marital Status			P=0.399	
Married	Reference			
Single	0.82 (0.53-1.27)		P=0.368	
Divorced/Separated	0.85 (0.47-1.52)		P=0.580	
Widowed	0.55 (0.28-1.08)		P=0.082	
Unknown	0.71 (0.33-1.52)		P=0.374	
Race			P=0.610	
White	Reference			
Black	0.76 (0.33-1.79)		P=0.533	
Other	0.80 (0.47-1.36)		P=0.414	
Primary Site				
Vertebral Column (C1-L5)	Reference			
Pelvic bones, sacrum, coccyx, and associated joints	1.95 (1.32-2.87)		**P<0.001**	
Disease Stage			**P<0.001**	
Localized	Reference			
Regional	2.00 (1.41-2.84)		P<0.001	
Distant	0.85 (0.47-1.54)		P=0.586	
Unstaged	0.19 (0.06-0.56)		P=0.003	
Histological Type			P=0.173	
Chordoma, NOS	Reference			
Chondroid chordoma	2.19 (0.77-6.21)		P=0.142	
Dedifferentiated chordoma	0.37 (0.07-2.00)		P=0.250	
Tumor Size			P=0.848	
<5 cm	Reference			
5-10 cm	1.07 (0.69-1.66)		P=0.752	
>10 cm	1.26 (0.73-2.16)		P=0.404	
Unknown	1.00 (0.61-1.66)		P=0.986	
Radiotherapy				
Yes	Reference			
No	1.39 (1.00-1.93)		P=0.051	
Chemotherapy				
Yes	Reference			
No	1.35 (0.59-3.06)		P=0.480	
Year of Diagnosis			P=0.502	
2000-2004	Reference			
2005-2009	1.36 (0.80-2.31)		P=0.250	
2010-2014	1.38 (0.83-2.28)		P=0.212	
2015-2019	1.46 (0.89-2.40)		P=0.138	

OR, odds ratio; CI, confidence interval; STR, subtotal resection; GTR, gross total resection; NOS, not otherwise specified.

Variables with statistical significance are shown in bold.

There was also no difference in the frequency of radiotherapy treatment across different age groups; 45.5% of patients aged 20-39 received radiotherapy compared to 48.3% of patients aged 80-99 (p=0.762; **Figure 2**). Additionally, when we stratified our patient cohort by extent of resection, age at diagnosis was not associated with receipt of radiotherapy for patients receiving no surgery (p=0.630), STR (p=0.262), or GTR (p=0.605) (**Table 4**). Moreover, in our multivariable logistic regression analysis with radiotherapy as the dependent variable, age at diagnosis was not predictive of receiving radiotherapy (p=0.414; **Table 5**). Variables that were predictive of receiving radiotherapy included primary site (p<0.001), disease stage (p=0.002), extent of resection (p=0.033), and year of diagnosis (p=0.020) (**Table 5**). Patients diagnosed with spinal chordoma between 2010-2014 (OR 2.02; 95% CI 1.27-3.01, p=0.003) and patients undergoing no surgery (OR 1.74; 95% CI 1.15-2.64, p=0.009) were more likely to receive radiotherapy (**Table 5**).

**Table 4:** Frequency of Radiotherapy Among Spinal Chordoma Patients Stratified By Extent of Resection.

Age at Diagnosis	20-39 (%)	40-59 (%)	60-79 (%)	80-99 (%)	P value
Extent of Resection					
No Surgery	11 (100%)	47 (100%)	96 (100%)	73 (100%)	P=0.630
Radiotherapy					
Yes	4 (36.4%)	21 (44.7%)	49 (51.0%)	39 (53.4%)	
No	7 (63.6%)	26 (55.3%)	47 (49.0%)	34 (46.6%)	
STR	39 (100%)	85 (100%)	119 (100%)	24 (100%)	P=0.262
Radiotherapy					
Yes	17 (43.6%)	50 (58.8%)	58 (48.7%)	10 (41.7%)	
No	22 (56.4%)	35 (41.2%)	61 (51.3%)	14 (58.3%)	
GTR	38 (100%)	100 (100%)	120 (100%)	19 (100%)	P=0.605
Radiotherapy					
Yes	19 (50.0%)	46 (46.0%)	48 (40.0%)	7 (36.8%)	
No	19 (50.0%)	54 (54.0%)	72 (60.0%)	12 (63.2%)	

P value <0.05 are shown in bold.

STR, subtotal resection; GTR, gross total resection.

Percentages may not add up to 100 because of rounding.

**Table 5:** Logistic Regression of Spinal Chordoma Patients (n=771), Demonstrating Clinical and Demographic Factors that were Predictive of Receiving Radiotherapy.

Characteristic	OR (95% CI)	P value
Age at Diagnosis		P=0.414
20-39	Reference	
40-59	1.19 (0.70-2.01)	P=0.519
60-79	0.91 (0.54-1.53)	P=0.715
80-99	0.81 (0.42-1.56)	P=0.523
Sex		
Male	Reference	
Female	0.88 (0.65-1.21)	P=0.436
Marital Status		P=0.281
Married	Reference	
Single	0.71 (0.47-1.08)	P=0.109
Divorced/Separated	0.96 (0.56-1.65)	P=0.884
Widowed	1.08 (0.62-1.89)	P=0.785
Unknown	0.55 (0.28-1.11)	P=0.094
Race		P=0.901
White	Reference	
Black	1.12 (0.50-2.52)	P=0.789
Other	0.92 (0.56-1.50)	P=0.727
Primary Site		
Vertebral Column	Reference	
Pelvic bones, sacrum, coccyx, and associated joints	0.42 (0.29-0.60)	**P<0.001**
Disease Stage		**P=0.002**
Localized	Reference	
Regional	1.32 (0.94-1.85)	P=0.109
Distant	1.24 (0.73-2.12)	P=0.435
Unstaged	0.29 (0.13-0.64)	P=0.002
Histological Type		P=0.718
Chondroid chordoma	Reference	
Conventional chordoma	1.54 (0.55-4.32)	P=0.416
Dedifferentiated chordoma	1.02 (0.26-3.97)	P=0.981
Tumor Size		P=0.490
<5 cm	Reference	
5-10 cm	0.95 (0.63-1.45)	P=0.822
>10 cm	1.33 (0.80-2.24)	P=0.276
Unknown	0.93 (0.59-1.48)	P=0.763
Extent of Resection		**P=0.033**
GTR	Reference	
STR	1.22 (0.85-1.77)	P=0.286
No Surgery	1.74 (1.15-2.64)	P=0.009
Chemotherapy	
Yes	Reference	
No	0.84 (0.42-1.71)	P=0.637
Year of Diagnosis		**P=0.020**
2000-2004	Reference	
2005-2009	1.27 (0.78-2.08)	P=0.331
2010-2014	2.02 (1.27-3.21)	P=0.003
2015-2019	1.53 (0.96-2.43)	P=0.074

OR, odds ratio; CI, confidence interval; STR, subtotal resection; GTR, gross total resection; NOS, not otherwise specified.

Variables with statistical significance are shown in bold.

### 3.2 Assessment of OS according to extent of resection

**Figure 3:**
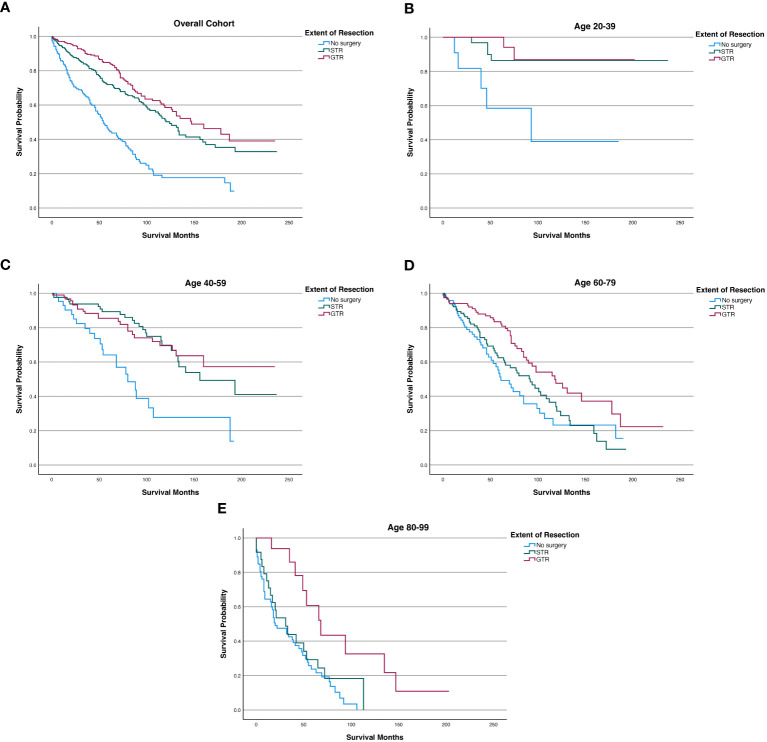
Kaplan-Meier survival curves in spinal chordoma patients based on extent of resection in the overall cohort and stratified by age. **(A)** Overall Cohort (P<0.001). **(B)** 20-39 years old (P=0.001). **(C)** 40-59 years old (P<0.001). **(D)** 60-79 years old (P=0.002). **(E)** 80-99 years old (P=0.002).

Among the overall patient cohort, our Kaplan-Meier analysis demonstrated that GTR was associated with greater OS than STR or no surgery (p<0.001; **Figure 3**; **Table 6**). The mean OS was 149.2 months for the GTR group, 132.7 months for the STR group, and 72.1 months for the no surgery group (**Table 6**). The 5-year OS and 10-year OS of patients in the GTR, STR, and no surgery groups were 84.8% and 58.6%, 71.9% and 50.3%, and 45.2% and 17.6%, respectively (**Table 6**).

**Table 6:** Kaplan-Meier analysis of overall survival for spinal chordoma patients in the overall cohort or stratified by age at diagnosis.

Characteristic	5-year, overall survival %	10-year, overall survival %	Mean overall survival (months)	Kaplan-Meier Log rank X^2^ test	P value
Overall cohort				88.74	**<0.001**
No Surgery	45.20%	17.60%	72.1		
STR	71.90%	50.30%	132.7		
GTR	84.80%	58.60%	149.2		
Age at diagnosis					
20-39				13.02	**0.001**
No surgery	58.40%	39.00%	102.8		
STR	86.40%	86.40%	210.8		
GTR	94.10%	86.90%	184.7		
40-59				20.59	**<0.001**
No surgery	64.10%	27.70%	95.7		
STR	89.30%	69.80%	163.3		
GTR	85.40%	69.50%	170.4		
60-79				12.12	**0.002**
No surgery	51.30%	23.20%	84.6		
STR	62.50%	31.30%	92.2		
GTR	83.40%	47.60%	128.5		
80-99				12.01	**0.002**
No surgery	23.70%	0.00%	35.2		
STR	29.20%	0.00%	43.3		
GTR	60.80%	32.60%	90.2		

Variables with statistical significance are shown in bold.

After stratifying our study cohort by age, our Kaplan-Meier analysis showed that extent of resection was significantly associated with OS for patients aged 20-39 (p=0.001), 40-59 (p<0.001), 60-79 (p=0.002), and 80-99 (p=0.002) (**Figure 3**). Among the 60-79 age cohort, patients receiving GTR had superior 5-year OS, 10-year OS, and mean OS (83.40%, 47.60%, and 128.5 months, respectively) compared to those of STR patients (62.50%, 31.30%, and 92.2 months) and no surgery patients (51.30%, 23.20%, and 84.6 months) (**Figure 3**; **Table 6**). Among the 80-99 age cohort, patients receiving GTR also had the highest 5-year OS, 10-year OS, and mean OS (60.80%, 32.60%, and 90.2 months, respectively) relative to those STR patients (29.20%, 0.00%, and 43.3 months) and no surgery patients (23.70%, 0.00%, and 35.2 months) (**Figure 3**; **Table 6**). In both the 40-59 and 20-39 age cohorts, STR and GTR patients had superior 5-year OS, 10-year OS, and mean OS compared to those of no surgery patients (**Figure 3**; **Table 6**).

### 3.3 Assessment of mortality risk according to extent of resection

To investigate whether extent of resection was a prognostic factor for mortality across different age groups, we first performed a univariate Cox regression analysis. The results showed that extent of resection was associated with decreased mortality for patients aged 20-39 (p=0.008), 40-59 (p<0.001), 60-79 (p=0.003), and 80-99 (p=0.005) (**Table 7**). After including age at diagnosis, sex, marital status, race, primary site, disease stage, histological type, tumor size, extent of resection, radiotherapy, chemotherapy, and year of diagnosis as covariates in our multivariate Cox regression analysis, extent of resection was a statistically significant prognostic factor of decreased mortality in patients aged 40-59 (p=0.002), 60-79 (p=0.012), and 80-99 (p<0.001) (**Table 7**). Specifically, patients receiving GTR aged 40-59 (HR=0.26, CI=0.12-0.55, p<0.001), 60-79 (HR=0.51, CI=0.32-0.82, p=0.005), and 80-99 (HR=0.14, CI=0.05-0.37, p<0.001) had a lower risk of mortality compared to patients undergoing no surgery (reference) (**Table 7**). For the 20-39 age cohort, we were unable to reliably evaluate the association between extent of resection and mortality risk in our multivariate analysis due to an insufficient sample size.

**Table 7:** Univariate and multivariate analyses for predicting mortality in spinal chordoma patients stratified by age at diagnosis.

Characteristic	Univariate Analysis		Multivariate Analysis	
	Hazard Ratio (95% CI)	P value	Hazard Ratio (95%CI)	P value
Age at diagnosis		**p=0.008**	
20-39				
No surgery	Reference			
STR	0.18 (0.05-0.66)	p=0.010		
GTR	0.12 (0.02-0.61)	p=0.011		
40-59		**p<0.001**		**p=0.002**
No surgery	Reference		Reference	
STR	0.33 (0.18-0.59)	p<0.001	0.37 (0.18-0.75)	p=0.006
GTR	0.32 (0.17-0.57)	p<0.001	0.26 (0.12-0.55)	p<0.001
60-79		**p=0.003**		**p=0.012**
No surgery	Reference		Reference	
STR	0.84 (0.57-1.25)	p=0.39	0.85 (0.54-1.33)	p=0.483
GTR	0.50 (0.33-0.76)	p=0.001	0.51 (0.32-0.82)	p=0.005
80-99		**p=0.005**		**p<0.001**
No surgery	Reference		Reference	
STR	0.79 (0.47-1.33)	p=0.366	0.48 (0.23-1.00)	p=0.050
GTR	0.28 (0.13-0.60)	p=0.001	0.14 (0.05-0.37)	p<0.001

STR, subtotal resection; GTR, gross total resection.

Variables with statistical significance are shown in bold.

## 4 Discussion

With the global expansion of the elderly population, neurosurgeons face an increasing probability of having to manage spinal chordoma patients of increasingly advanced ages. Although several studies have explored the impact of extent of resection on local recurrence rates and survival outcomes in patients with spinal chordoma, few studies contain elderly patients of sufficient sample size. As such, it remains unclear whether geriatric patients can benefit from aggressive surgical resection, particularly when these operations remain highly invasive in a population with limited life expectancy and complex medical comorbidities.

In this large, population-based study, we comprehensively analyzed treatment patterns and survival outcomes of adult patients with spinal chordoma across different age groups. Our multivariate analysis indicated that middle-aged and elderly patients who received GTR had a significantly lower risk of mortality compared to patients who received no surgery. While univariate analysis revealed a significant association between surgery and OS for young adult patients aged 20-39 (p=0.008), we were unable to determine significance on multivariate analysis due to the small sample size in the young adult subgroup. Enrollment of more patients may yield a significant association between surgery and OS in young adults after adjusting for sociodemographic and clinical covariates. Although patients in all age subgroups demonstrated improved survival with GTR, the relative survival benefit of GTR was most pronounced for patients aged 80-99, with over twice the mean OS of patients who undergo STR (90.2 vs. 43.3 months) and nearly triple the mean OS of patients who receive no surgery (90.2 vs. 35.2 months). Together, this data suggests that patients with spinal chordoma should not be excluded from GTR on the basis of age alone.

Current guidelines for the treatment of spinal chordomas published by the Chordoma Foundation in 2015 recommend GTR with adjuvant radiotherapy when feasible (20). However, the term “when feasible” is ambiguous and subject to individual provider interpretation, particularly as it pertains to elderly patients. Certain factors are known to preclude the achievement of negative margins, including large paraspinal masses, high-grade spinal cord compression, risk of postoperative morbidity, and bilateral vertebral artery invasion (21–23). The feasibility of GTR also depends on patients’ overall health. Elderly patients tend to exhibit a decline in physiological function, inferior bone quality, and spinal degeneration, coupled with chronic comorbidities, such as diabetes and cardiovascular disease, making them poor surgical candidates (24, 25). Moreover, elderly patients may lack sufficient social support to assist with recovery following GTR. Thus, the results of our study should be interpreted in the context of the numerous factors that go into patient selection. We observed a decrease in the frequency of GTR with increasing age [from 43.2% (20-39 years) to 16.4% (80-99 years); p<0.001], with similar results in our multivariable logistic regression analysis with GTR as the dependent variable. The decreased frequency of GTR as a function of age likely reflects the appropriate exclusion of patients with considerable risks for peri- and postoperative complications in addition to patient preferences. However, it is also likely that patients who are suitable candidates for surgery are denied treatment due to the paucity of data on geriatric outcomes following GTR, and our study fills this knowledge gap in the current literature.

Aggressive surgical cytoreduction in elderly patients must take into account both oncological and functional outcomes. The former can be approximated by tumor residue after surgical resection, which predicts local recurrence rates and OS (6). The latter focuses on the development of major functional sequelae, which affects patients’ health-related quality of life (QoL). Gait disturbance, bladder/fecal incontinence, sexual impotence, and spinal instability are all potential consequences following sacral amputation depending on the nerve roots sacrificed in order to obtain negative margins. However, these are also possible symptoms of chordoma as a result of tumor progression. Moran et al. noted that there were no significant changes in preoperative and 6 months postoperative bladder, bowel, or motor function for patients undergoing en bloc resection of primary sacral tumors (26). Moreover, Schwab et al. compared the QoL among 12 middle-aged spinal chordoma patients (45-65 years) before and after surgery and reported no significant difference besides higher rates of anxiety post-treatment (27). This suggests that patients may have minimal changes in QoL following resection, but with the added benefit of improved survival. However, further comprehensive studies are needed to evaluate the QoL of geriatric patients before and after spinal chordoma resection. For patients who do not accept the functional sequelae associated with GTR, STR can serve as an alternative option. In our Kaplan-Meier analysis, STR was associated with an improvement in survival relative to no surgery for patients aged 40-59 (163.3 vs. 95.7 months, p<0.001), 60-79 (92.2 vs. 84.6 months, p=0.002), and 80-99 (43.3 vs. 35.2 months, p=0.002). In addition, Fontes and Toole performed STR in an octogenarian with spinal chordoma and observed improved QoL during follow-up at 13 months, further implicating the use of surgery in carefully selected patients (16).

The role of radiation therapy, either as adjuvants or standalone options, in the management of advanced progressive chordoma has expanded in the past 10 years, which may be especially attractive for elderly patients (28–30). In our study, we found that elderly patients receive radiotherapy at similar rates as younger patients despite being less likely to receive surgery, connoting that elderly patients are more likely to be treated with radiation alone. In an analysis of 345 sacral chordoma patients using SEER 1973-2011 data, Yu et al. concluded that the use of radiation in the treatment of chordoma surprisingly decreased over time (31). However, we included a larger patient cohort (771 patients) and utilized more recent SEER data (2000–2019) and observed an increase in the usage of radiation over time. Specifically, patients diagnosed between 2010 and 2014 were more likely (OR=2.02, CI=1.27-3.21, p=0.003) to receive radiotherapy compared to patients diagnosed between 2000 and 2004. This finding coincides with the passage of the Affordable Care Act in 2010, which increased health insurance coverage among the general populace, and may have allowed more patients to afford treatment with multiple modalities (i.e. surgery and radiotherapy) (32). Alternatively, novel advances in specialized radiation modalities (e.g. proton beam therapy, stereotactic radiosurgery, and intensity modulated radiation therapy) may have translated into the more widespread usage of radiotherapy (28). However, the likelihood of receiving GTR remained stable over the duration of our study period, suggesting that the growth of radiotherapy is not displacing GTR.

Taken together, the results of our study do not suggest that all elderly patients with spinal chordoma should receive GTR. A comprehensive assessment and rigid screening are required in order to select patients and should involve a conglomeration of factors, including the severity of a patient’s symptoms, the impact of these symptoms on function and QoL, the overall life expectancy of the patient, the functional status and underlying comorbidities of the patient, as well as the patient’s goals of care. As part of effective patient education and surgical counseling, the physician should be able to explain risks of surgery to the patient as well as expected survival benefits. Previously, physicians were forced to extrapolate findings from clinical studies involving younger, healthier subjects, which is dangerous because older patients may be more likely to experience cancer-related morbidity and mortality from under-treatment. Instead, we show that advanced age by itself should not restrict patients from receiving GTR.

The SEER national registry is one of the largest publicly accessible sources of patient outcome data; however, there are limitations to the retrospective design of our study. First, the SEER database does not collect specific information on patient characteristics such as performance status or medical comorbidities, leading to potential selection bias among the patients that received operative intervention. Thus, we could not assess whether the extent of resection was an independent predictor of mortality in our age-stratified multivariate Cox regression analysis. Moreover, the SEER database does not distinguish between primary and locally recurrent chordomas. The inclusion of recurrent chordomas underestimates the survival outcomes for primary tumors. Furthermore, different surgical approaches (i.e. total en bloc spondylectomy with posterior only or combined posterior/antero-lateral approaches) have varying risks of great vessel injury which could affect OS, but this information is not contained in the SEER database. Likewise, the SEER database does not distinguish spinal chordomas located in the cervical spine or thoracolumbar spine. Resection of cervical chordomas carry more operative risk than for chordomas at other spinal levels. Without knowing the proportion of patients with cervical or thoracolumbar chordomas that received surgical treatment, we could not control for this confounder. Moreover, pertinent details on chemotherapeutic regimens or radiotherapy such as the modality, dosage, or beam energy are not documented in the SEER database, which could further bias our results. Thus, we could not distinguish whether patients in the no surgery group that received radiotherapy were treated with palliative or therapeutic regimens. As a result, we could not ascertain whether GTR or STR truly offers a survival benefit over no surgery, but our data does show that elderly patients who were indicated for and received surgery in our study had better survival than elderly patients who were not indicated for or did not receive surgery. It is plausible that elderly patients treated with STR or GTR may have similar survival to elderly patients treated with no surgery and therapeutic doses of radiotherapy when various sociodemographic and clinical factors are controlled for. However, our study represents data captured in a population-based cohort over a twenty-year period and would be useful in the neurosurgical literature. Additionally, the lack of data on peri- and postoperative infectious, mechanical, and hemorrhagic complications reduces the granularity of our results as these rates are often instrumental in the calculation of the risk to benefit ratio for surgery. Lastly, the SEER database does not include local recurrence data, so we were limited to OS as our primary study outcome.

## 5 Conclusion

In this large, population-based study, we observed a clear association between receiving GTR and improved survival for elderly patients with spinal chordoma. Advanced age in itself should not be a contraindication to GTR when indicated. The optimal treatment strategy for elderly patients with spinal chordoma should take into consideration patient preferences and include a detailed assessment by a multidisciplinary team of physicians. Future studies evaluating QoL and complication rates in elderly patients undergoing GTR are warranted.

## Data availability statement

Publicly available datasets were analyzed in this study. This data can be found here: https://seer.cancer.gov/data/access.html, SEER Research Plus Dataset, 17 Registries (2000–2019).

## Ethics statement

Ethical approval was not required for the study involving humans in accordance with the local legislation and institutional requirements. Written informed consent to participate in this study was not required from the participants or the participants’ legal guardians/next of kin in accordance with the national legislation and the institutional requirements.

## Author contributions

JP: Conceptualization, Data curation, Formal analysis, Methodology, Software, Writing – original draft. ES: Conceptualization, Data curation, Formal analysis, Methodology, Software, Writing – original draft. BR: Data curation, Formal analysis, Methodology, Software, Writing – original draft. AK: Data curation, Formal analysis, Methodology, Software, Writing – original draft. EO: Data curation, Formal analysis, Methodology, Software, Visualization, Writing – original draft. KP: Data curation, Formal analysis, Methodology, Software, Visualization, Writing – original draft. TN: Conceptualization, Supervision, Writing – review & editing. PS: Conceptualization, Supervision, Writing – review & editing. ZG: Conceptualization, Supervision, Writing – review & editing.
