# Supplementary material for: Gross total resection and survival outcomes in elderly patients with spinal chordoma: a SEER-based analysis
Source: Front Oncol. 2024 Jan 30;13:1327330. doi: 10.3389/fonc.2023.1327330 (PMC10862492; doi:10.3389/fonc.2023.1327330)
Supplement: Supplementary file 1 [file Table_1.docx]

| **Supplementary Table S.1.** Criteria for determination of the extent of resection | | | |
| --- | --- | --- | --- |
| **Surgery Code** | **Description** | **Extent of Resection** | **N** |
| 00 | None; no surgery of primary site; autopsy only | No Surgery | 227 |
| 15 | Local tumor destruction | Subtotal Resection | 4 |
| 19 | Local tumor destruction or excision, NOS | Subtotal Resection | 13 |
| 25 | Local excision | Subtotal Resection | 147 |
| 26 | Partial resection | Subtotal Resection | 103 |
| 30 | Radical excision or resection of lesion WITH limb salvage | Gross Total Resection | 260 |
| 40 | Amputation of limb | Gross Total Resection | 4 |
| 41 | Partial amputation of limb | Gross Total Resection | 3 |
| 42 | Total amputation of limb | Gross Total Resection | 2 |
| 50 | Major amputation, NOS | Gross Total Resection | 3 |
| 51 | Forequarter, including scapula | Gross Total Resection | 0 |
| 52 | Hindquarter, including ilium/hip bone | Gross Total Resection | 2 |
| 53 | Hemipelvectomy, NOS | Gross Total Resection | 2 |
| 54 | Internal hemipelvectomy | Gross Total Resection | 1 |
| 90 | Surgery, NOS | Excluded | 24 |
| 99 | Unknown if surgery performed; death certificate ONLY | Excluded | 11 |
| The content of this table was adapted from the SEER Program and Staging Manual 2018 (Appendix C: Surgery Codes for Site Specific Coding Modules C40.0-C41.9) | | | |

The SEER database uses the surgery codes and definitions listed in supplementary table S1 above for all bone tumors regardless of location (e.g. upper limb, lower limb, skull base, spine, ribs, etc.). Thus, a sacral amputation may be coded as “amputation of limb” even though no anatomic limb was amputated. In this instance, “limb” refers to the primary site specified, which in the present study is the spine. Final SEER surgery code classifications are based on both operative and pathology reports when available. The classifications used in our study are consistent with previous SEER-based spinal chordoma studies.(18-19)
